# Association of High Expression of Gro*β* with Clinical and Pathological Characteristics of Unfavorable Prognosis in Gastrointestinal Stromal Tumors

**DOI:** 10.1155/2015/171035

**Published:** 2015-04-07

**Authors:** Hui Zhao, Huijun Zhu, Qin Jin, Shu Zhang, Wei Wang, Defeng Wang, Jianfei Huang

**Affiliations:** ^1^Department of Interventional Radiology, Nantong University Affiliated Hospital, Nantong, Jiangsu 22601, China; ^2^Department of Pathology, Nantong University Affiliated Hospital, Nantong, Jiangsu 22601, China; ^3^Department of Science and Technology Branch, Nantong University Affiliated Hospital, Nantong, Jiangsu 22601, China

## Abstract

GRO*β* (CXCL2) is a chemokine produced by endotoxin-treated macrophages that mediates inflammation and tumor development. However, little is known about GRO*β* expression in gastrointestinal stromal tumors (GIST) or the relationship between GRO*β* expression and clinical attributes of GIST. GRO*β* expression was examined via immunohistochemical staining of 173 GIST samples using tissue microarray. The relationship between GRO*β* expression and relevant patient and tumor characteristics was assessed, using chi-square tests. Univariate and multivariate analysis was carried out using the Cox regression method. High GRO*β* cytoplasm staining was detected in 56 (32.4%) specimens; high GRO*β* nuclear staining was detected in 64 (37.0%) specimens. High GRO*β* cytoplasm staining was significantly associated with patients' age (*P* = 0.043) and tumor location (*P* = 0.014), while high GRO*β* nucleus staining was significantly associated with mitotic index (*P* = 0.034), tumor location (*P* = 0.049), and AFIP-Miettinen risk classification (*P* = 0.048). Kaplan-Meier survival curves showed GIST patients with low GRO*β* cytoplasm expression (*P* = 0.023) and mitotic index < 6 per 50 HPFs (*P* = 0.026) to have a more favorable prognosis. These findings indicate that GRO*β* expression correlates with malignant GIST phenotypes and could be an unfavorable prognostic marker in patients with GIST.

## 1. Introduction

Gastrointestinal stromal tumor (GIST) is an uncommon type of mesenchymal neoplasm primarily originating from the wall of the stomach, small intestine, and colon [1]. The mean annual incidence of GIST is 10–15 cases per million people, affecting mainly older people with a median age of 58 years [2–5]; however, GISTs have also been observed in the pediatric population [6]. GISTs can be recognized immunohistochemically by CD117, the 145 kDa transmembrane glycoprotein KIT, and CD34 proteins [7–9]. They are believed to arise from the interstitial cells of Cajal (ICC) or from interstitial mesenchymal precursor stem cells [10, 11]. GIST development is usually expansive and the primary metastatic paths are hematogenous and seeding metastasis, which makes GIST unique [12]. The clinical characteristics of GIST vary depending on the location, size, and aggressiveness of the tumor [13]. The most common symptoms are bleeding from the upper gastrointestinal tract and abdominal pain; however, many GIST patients remain asymptomatic and are discovered only incidentally [14].

For now, radical surgery is the predominant treatment for primary resectable GIST; however, GIST often recurs; nearly 50% of GIST patients with curative resections develop recurrence or metastasis. Neither classic cytotoxic chemotherapy nor radiotherapy is reliably efficacious in managing GIST; hence the prognosis of patients with unresectable or metastatic GIST is poor [15, 16]. Imatinib (IM), an oral 2-phenylaminopyrimidine derivative that selectively stabilizes certain tyrosine kinases in the inactivated form and prevents their constitutive autophosphorylation has revolutionized GIST therapy and significantly improved clinical outcomes of patients with advanced GIST [17, 18]. IM has become the standard treatment for metastatic or unresectable GIST, leading to objective responses or stable disease of ≥80% and median time to progression of up to 2 years [19]. However, the effectiveness of this novel targeted therapy can vary depending on tumor location, tumor size, histological risk stratification, and mutation status of the receptor tyrosine kinase [20]. Therefore, identifying biomarkers that can guide molecular-targeted therapy for GIST patients is important.

GRO*β* (CXCL2) belongs to the growth-related oncogene (GRO) subgroup of chemokines, which act as specific modulators in leukocyte migration to sites of inflammation and are also involved in the development and progression of carcinogenesis [21]. GRO*β* was first identified from cell culture supernatants of melanoma cells and believed to partially mediate inflammation [22]. A growing number of studies have focused on the relationship between GRO*β* and cancers. Compared with normal controls, Dong et al. reported that higher levels of GRO*β* could be detected in esophageal squamous cell carcinoma patients [23]. They also showed that GRO*β* and its downstream product early growth response protein (EGR1) were associated with cisplatin-induced apoptosis in a human esophageal squamous cell carcinoma cell line [24]. A study using a melanoma tumor model elucidated the function of GRO*β* in mediating tumor angiogenesis and found GRO*β* to be highly expressed in melanoma tumors. Transfection of GRO*β* into immortalized nononcogenic cells gave them the ability to form tumors [25, 26]. GRO*β* is reportedly upregulated in ER*α*-breast cancer patients and correlates with shorter relapse-free survival [27]. Although there are histological discrepancies of tumor origin in esophageal cancer, melanoma, and breast cancer, GRO*β* critically showed its potential oncogenic characteristics. However, the relationship between GRO*β* expression and clinicopathological features, especially prognosis, has been barely investigated.

In this present study, the GRO*β* protein expression was investigated in a number of GIST samples with tissue microarrays (TMAs), using immunohistochemistry (IHC) analysis. Moreover, the association between GRO*β* expression and the clinicopathological attributes was examined in GIST patients. Finally, the prognostic significance of GRO*β* protein expression level in GIST was evaluated.

## 2. Materials and Methods

### 2.1. Collection of Patient Samples

In this study, we enrolled 173 patients with GIST who had been hospitalized in the Nanjing First Hospital Affiliated to Nanjing Medical University and the Affiliated Hospital of Nantong University between 2003 and 2010. Diagnosis was based on histopathological appearance that was compatible with GIST and was confirmed by positive IHC staining for c-KIT. Original clinical data were collected, including patient age, tumor size, mitotic index, growth pattern, tumor location, tumor risk classification [28, 29], and 5-year and 10-year overall survival (OS). Potential risk classification for malignancy was evaluated using AFIP-Miettinen risk classification criteria [28, 29]. None of the patients received preoperative radiotherapy or chemotherapy or tyrosine-kinase inhibitor treatment. Each patient gave written informed consent for publication of this study and the protocol of this research was approved by the Human Research Ethics Committee of each hospital.

### 2.2. TMAs Construction and IHC Analysis

A total of 173 formalin-fixed, paraffin-embedded GIST tissues, collected between 2003 and 2010, were obtained from the Nanjing First Hospital Affiliated to Nanjing Medical University and Affiliated Hospital of Nantong University. Representative 2.0 mm tissue core samples from each GIST patient were subjected to TMA using Tissue Microarray System (Quick-Ray, UT06, UNITMA, Korea) as described [30] in the Department of Clinical Pathology, Nantong University Hospital, Jiangsu, China.

We performed IHC analysis to evaluate protein expression of GRO*β* in GIST. Briefly, paraffin tissue sections (4 *μ*m) were deparaffinized in 100% xylene and rehydrated in a descending ethanol series according to standard protocols. The TMA sections were incubated for 1 hour with primary rabbit anti-GRO*β* polyclonal antibody (1.0 *μ*g/mL, PeproTech, USA) in Tris-buffered saline (TBS) containing 1% bovine serum albumin (BSA), washed, and incubated with horseradish peroxidase-conjugated antibody using EnvisionTM kit (K5007, Dako, USA). As a negative control, phosphate-buffered saline (PBS) was employed instead of the primary antibody. GRO*β* immunostaining was scored by blinded observers according to intensity and percentage of GRO*β* ^+^ cells. Staining intensity was scored according to four grades: 0, 1, 2, or 3, ranging from negative and weak to strong intensity. The percentage of GRO*β* ^+^ cells was scored as follows: 0 for 0%, 1 for 1–33%, 2 for 34–66%, and 3 for 67–100% [31]. The product of the percentage and intensity scores was used as the final staining score as described previously.

The cutoff point for the GRO*β* expression score that was statistically significant in terms of OS was set using the X-tile software program (the Rimm Lab at Yale University; http://www.tissuearray.org/rimmlab/) as described elsewhere [32]. The degree of cytoplasmic GRO*β* staining was quantified using a two-level grading system, and staining scores were defined as follows: 0–4, low and none expression and 5–9, high expression. The level of nuclear GRO*β* ^+^ staining was also divided into two grades and confirmed that low and none expression was 0–2 and high expression was 3–9.

### 2.3. Statistical Analysis

Relationships between GRO*β* expression and clinicopathological attributes were analyzed by *χ* ^2^ tests. Survival rates were calculated by the Kaplan-Meier method and compared by log rank test. Univariate and multivariate analyses used a Cox proportional hazards regression model. All statistical analyses used SPSS 18.0 statistic software (SPSS Inc, Chicago, IL) and STATA 12.0 (StataCorp, College Station, TX, USA).

## 3. Results

### 3.1. Clinical Characteristics of GISTs

We enrolled 173 patients with GIST and median age 57.8 years (range: 38–81 years) in this study. Of them, 89 (51.4%) patients were ≤60 years of age while 69 (39.9%) were older; 42 (24.3%) had tumors <5 cm in diameter, 72 (41.6%) had tumors 5–10 cm in diameter, and 37 (21.4%) had tumors >10 cm in diameter. For mitotic index, 64 (37.0%) patients had 0–5/50 HPFs, and 80 (46.2%) had >6/50 HPFs. Of those patients, 136 (78.6%) had single nodules, while 19 (11.0%) had multiple nodules; 75 (43.4%) patients had tumors in the stomach, 61 (35.5%) in the intestines, and 22 (12.7%) in other organs. The AFIP-Miettinen risk classifications of 104 patients were very low to low risk and 39 were moderate to high risk.

### 3.2. Expression and Localization of GRO*β* in GISTs

**Figure 1:**
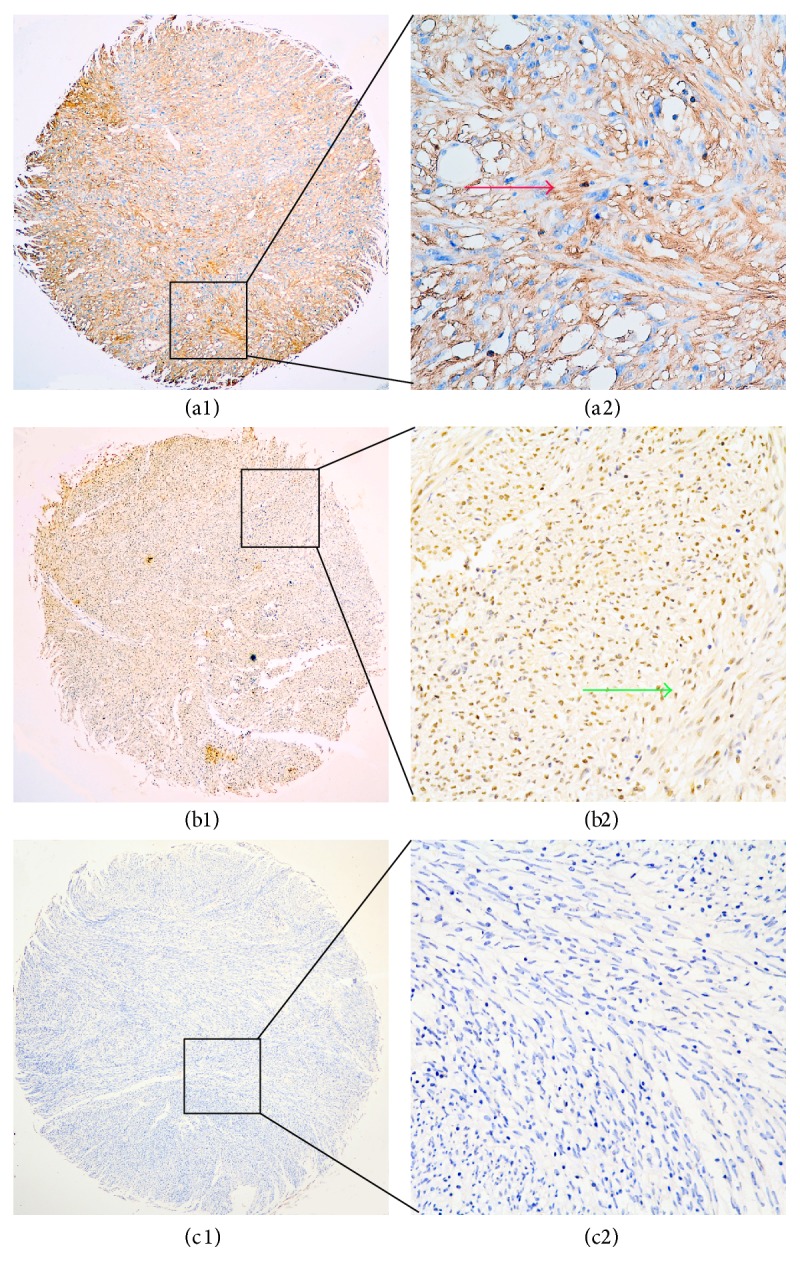
Representative patterns of GRO*β* expression in gastrointestinal stromal tumors (GIST) tissues. ((a1), (a2)) Strong GRO*β* cytoplasm expression (red arrow) was detected in GIST, with GRO*β* ^−^ immunohistochemical staining in nucleus (blue). ((b1), (b2)) Strong GRO*β* nucleus expression (green arrow) was detected in GIST. ((c1), (c2)) Expression of GRO*β* protein was negative in cytoplasm and nucleus of GIST tissue. Original magnifications: (a1), (b1), and (c1): 10x; (a2), (b2), and (c2): 40x.

We examined the expression of GRO*β* in GISTs TMAs using IHC analysis. Representative immunohistochemical GRO*β* staining patterns are shown in Figure 1. GRO*β* ^+^ staining was predominantly localized to the cytoplasm and nucleus of tumor cells. All tissue samples were scored and categorized according to the cutoff point for GRO*β* expression determined using the X-tile software program. In tumor cells, high GRO*β* protein staining with the cytoplasm was detected in 56 of 173 (32.4%) GIST tissues; the other 117 samples showed low or no GRO*β* protein staining with the cytoplasm. Similarly, high GRO*β* protein staining with the nucleus was detected in 64 of 173 (37.0%) GIST tissues and the other 109 showed low or no GRO*β* protein staining with the nucleus. Moreover, high GRO*β* protein staining with the cytoplasm and nucleus was observed in 30 of 173 (17.3%) GIST cases while low or no GRO*β* protein staining with the cytoplasm and nucleus was observed in 84 of 173 (48.6%) GIST cases (Table 1).

### 3.3. Relationship between GRO*β* Expression and Clinicopathological Attributes

The relationship between GRO*β* expression and the clinicopathological attributes of the 173 patients is shown in Table 2. High GRO*β* positive staining within the cytoplasm was significantly associated with patients' age (*P* = 0.043) and tumor location (*P* = 0.014). High GRO*β* positive staining within the nucleus was significantly associated with mitotic index (*P* = 0.034), tumor location (*P* = 0.049), and AFIP-Miettinen risk classification (*P* = 0.048). In contrast, GRO*β* expression was not associated with any other clinical parameters, including tumor size and growth pattern (Table 2).

### 3.4. Survival Analysis

**Figure 2:**
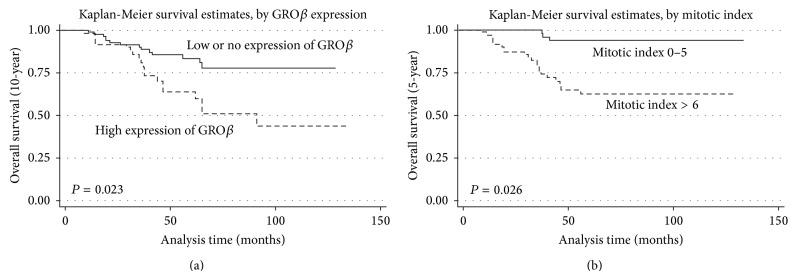
Kaplan-Meier analysis of the relationship between clinicopathologic factors and overall survival of gastrointestinal stromal tumors (GIST) patients. Overall survival was significantly longer in patients with (a) low (solid line) versus high GRO*β* expression (dash line) and those with (b) small (solid line) versus large mitotic indices (dash line).

Univariate analyses showed that increased expression of GRO*β* in both cytoplasm and nucleus, tumor size, mitotic index, and AFIP-Miettinen risk classification was associated with the prognosis of GIST patients for 5-year and 10-year overall survival rates (all *P* < 0.05, Table 3). Multivariate analyses further indicated that cytoplasm expression of GRO*β*  (*P* = 0.023) and mitotic index (*P* = 0.026) were significantly correlated with 5-year and 10-year overall survival rates, respectively (Table 3). Kaplan-Meier survival curves showed that GIST patients with low GRO*β* cytoplasm expression and mitotic index >6 per 50 HPFs had more favorable prognosis (Figures 2(a) and 2(b)).

## 4. Discussion

Chemokines are classified by their amino acid composition, functional activity, and receptor binding properties. They include four subfamilies that are characterized by the first two of four conserved cysteine residues (i) C, (ii) CC, (iii) CXC, and (iv) CXXXC [26]. The CXC chemokine family is also composed of two subtypes, ELR^+^ and ELR^−^, identified by a particular Glu-Leu-Arg (ELR) motif preceding the first cysteine residue [33]. CXC chemokines containing this ELR motif have been suggested to act as angiogenic factors that can stimulate endothelial cell migration, which could be regarded as tumor-promoting [34]. GRO*β* belongs to ELR^+^ subgroup of CXC-chemokines and several studies have reported the relationship between GRO*β* and human cancers [23–27]. There are at least five critical roles in which CXC chemokines play in the tumor microenvironment: (a) modulating leukocyte infiltration ability, (b) modifying tumor immune response, (c) regulating angiogenesis, (d) acting as growth and survival factors, and (e) managing the movement of tumor cells [35]. Furthermore, CXC chemokine receptor 2 (CXCR2) is a member of the seven-transmembrane domain rhodopsin-like G protein-coupled receptors, which modulates functions of ELR^+^ CXC chemokines such as angiogenesis [36]. CXCR2 is well known for playing critical roles in cancer progression by modulating the cell cycle, apoptosis, and angiogenesis [37, 38]. Although CXCR2 has a high affinity for Interleukin-8 (IL-8) and GRO*α* and a low affinity for GRO*β*, GRO*β*/CXCR2 loops may play an important role in the development and maintenance of certain type of human cancer, involving activation of NF-*κ*B and other possible signaling pathways, including RANKL, AKT, STAT3, and MAPK [39, 40]. In our previous research, high expression of CXCR2 was detected in human laryngeal squamous cell carcinoma and was significantly associated with its poor prognosis [41]. Moreover, it is reported that GRO*β* enhances transcription of EGR-1, via the extracellular signal-regulated kinase 1/2 (ERK1/2) pathway. Wang et al. stated that GRO*β* signaling was mediated through ERK1/2 and that blocking GRO*β* substantially inhibited signaling through ERK1/2. GRO*β* contributed dramatically to the elevated transcription of EGR-1 in cultured cancer cells [40]. As EGR-1 is known to affect the expression of many genes involved in tumorigenesis, including IGF-II and VEGF [42, 43], the relationship between GRO*β* and EGR-1 may imply the significant function of GRO*β* in tumorigenesis. In all, although the exact role of GRO*β* in GIST is not well elucidated, it appears rationale to assume that the GRO*β*/CXCR2 loop may affect GIST pathophysiology. In this present study, the clinicopathological attributes of GRO*β* in GIST were explored, particularly the relationship between GRO*β* expression and GIST prognosis.

By IHC analysis, we found GRO*β* overexpression not only in cytoplasm of tumor cells, but also in their nuclei. Specifically, 32.4% of GIST patients exhibited cytoplasmic staining of GRO*β* while 37.0% exhibited nucleus staining of GRO*β*. Although it was reported that GRO*β* expression located in cytoplasm of cancer cells [40], we observed the nuclei mode of GRO*β* expression. In our previous research, similar scenario occurred in which positive staining for a proliferation inducing ligand (APRIL) was witnessed in both cytoplasm and nucleus of GIST cells [31]. Moreover, slight nuclei expression of GRO*β* was also reported in oral squamous cell carcinoma [22]. Hence the different location of GRO*β* expression is rational and may be attributed to differences in tumor types, antibodies used, or experimental methods. In this present research, high cytoplasmic GRO*β* staining was associated with the pathologic characteristics of patients' age and tumor location, and high nuclear GRO*β* staining was associated with mitotic index, tumor location, and AFIP-Miettinen risk classification. Absence of control groups, such as adjacent noncancerous tissue, is a deficiency of our study. However, the localization of GIST makes it difficult to collect nontumor tissue samples. Therefore, our IHC analysis used negative expression of GRO*β* in whole TMA section as an internal control which was used to analyze and compare with other samples as having high or low GRO*β* expression.

Univariate analysis indicated that low GRO*β* expression (both cytoplasmic staining and nuclear staining) correlated with favorable prognosis for GIST patients, as determined by both 5-year and 10-year survival rates. Multivariate analysis further confirmed that high GRO*β* cytoplasmic staining and small mitotic index independently predicted poor prognosis in GIST patients (for 10-year and 5-year overall survival, resp.). These results are consistent with findings that showed that upregulated GRO*β* expression was detected in patients with breast cancer and indicated as poor prognosis [27].

Interestingly, other studies, inconsistently with ours, have reported that GRO*β* expression was downregulated in cancer and GRO*β* expression inhibited tumor formation [44, 45]. These conflicting results may be attributed to differences in tumor type, pathological samples, antibodies, or experimental methods. Further studies of the mechanisms of GRO*β* activity in tumorigenesis are necessary and of great interest.

To our knowledge, it is the first study to analyze GRO*β* expression in view of risk criteria and clinical behavior in GIST. Our results showed that high GRO*β* expression correlates with an aggressive malignant phenotype of GIST and GRO*β* could be identified as a novel prognostic marker for GIST.

## Figures and Tables

**Table 1:** Expression of GRO*β* in 173 GISTs with IHC analysis.

	Cytoplasm (%)	Nucleus (%)	Cytoplasm + nucleus (%)
High GRO*β* expression	56 (32.4%)	64 (37.0%)	30 (17.3%)
Low or no GRO*β* expression	117 (67.6%)	109 (63.0%)	84 (48.6%)

**Table 2:** Association of GRO*β* expression with clinical characteristics of GIST.

Groups	Number	Cytoplasm staining of GRO*β*				Nucleus staining of GRO*β*			
		Low/0 (%)	High (%)	Pearson *X* ^2^	*P*	Low/0 (%)	High (%)	Pearson *X* ^2^	*P*
Total	173	117 (67.6)	56 (32.4)			109 (63.0)	64 (37.0)		
Age									
≤60 years	89	52 (58.4)	37 (41.6)	4.108	0.043^*^	49 (55.1)	40 (44.9)	2.185	0.139
>60 years	69	51 (73.9)	18 (26.1)			46 (66.7)	23 (33.3)		
Unknown	15	14 (93.3)	1 (6.7)			14 (93.3)	1 (6.7)		
Tumor size									
<5 cm	42	31 (73.8)	11 (26.2)	1.529	0.466	27 (64.3)	15 (35.7)	3.798	0.150
5–10 cm	72	46 (63.9)	26 (36.1)			46 (63.9)	26 (36.1)		
≥10 cm	37	23 (62.2)	14 (37.8)			17 (45.9)	20 (54.1)		
Unknown	22	17 (77.3)	5 (22.7)			19 (86.4)	3 (13.6)		
Mitotic index (per 50 HPFs)									
0–5	64	45 (70.3)	19 (29.7)	1.288	0.256	44 (68.7)	20 (31.3)	4.502	0.034^*^
>6	80	49 (61.2)	31 (38.8)			41 (51.2)	39 (48.8)		
Unknown	29	23 (79.3)	6 (20.7)			24 (82.8)	5 (17.2)		
Growth pattern									
Single nodule	136	89 (65.4)	47 (34.6)	0.038	0.845	85 (62.5)	51 (37.5)	1.599	0.206
Multiple	19	12 (63.2)	7 (36.8)			9 (47.4)	10 (52.6)		
Unknown	18	16 (88.9)	2 (11.1)			15 (83.3)	3 (16.7)		
Location									
Stomach	75	58 (77.2)	17 (22.7)	8.588	0.014^*^	52 (69.3)	23 (30.7)	6.035	0.049^*^
Intestine	61	33 (54.1)	28 (45.9)			36 (59.0)	25 (41.0)		
Others	22	13 (59.1)	9 (40.9)			9 (40.9)	13 (59.1)		
Unknown	15	13 (86.7)	2 (13.2)			12 (80.0)	3 (20.0)		
AFIP-Miettinen risk classification									
Very low-low risk	104	72 (69.2)	32 (30.8)	2.952	0.086	67 (64.4)	37 (35.6)	3.927	0.048^*^
Moderate-high risk	39	21 (53.8)	18 (46.2)			18 (46.1)	21 (53.9)		
Unknown	30	24 (80.0)	6 (20.0)			24 (80.0)	6 (20.0)		

^*^ *P* < 0.05; HPF: high-power field.

**Table 3:** Univariate and multivariable analysis of prognostic factors in GIST for 5-year and 10-year survival.

Variable	Years	Univariate analysis			Multivariate analysis		
		HR	*P* value	95% CI	HR	*P* value	95% CI
Cytoplasmic expression of GRO*β*							
High versus low	10	2.521	0.009^*^	1.263–5.033	2.479	0.023^*^	1.135–5.418
	5	2.290	0.033^*^	1.071–4.895	1.927	0.133	0.819–4.537
Nuclear expression of GRO*β*							
High versus low	10	3.280	0.001^*^	1.611–6.677	2.082	0.071	0.939–4.619
	5	3.637	0.002^*^	1.631–8.110	2.039	0.111	0.849–4.898
Age (years)							
≤60 versus >60	10	1.215	0.583	0.606–2.435			
	5	1.080	0.841	0.507–2.300			
Tumor size (cm)							
	10	2.265	0.002^*^	1.360–3.772	3.182	0.076	0.884–11.448
	5	1.992	0.015^*^	1.145–3.466	3.559	0.098	0.792-15.982
Mitotic index (per 50 HPFs)							
0–5 versus >6	10	3.038	0.001^*^	1.869–4.937	2.695	0.095	0.842–8.632
	5	3.737	0.001^*^	2.117–6.598	6.086	0.026^*^	1.240–29.869
Growth pattern							
Single versus multiple	10	1.381	0.475	0.569–3.349			
	5	1.472	0.435	0.557–3.888			
Tumor location							
Stomach versus intestine versus others	10	1.357	0.218	0.835–2.203			
	5	1.320	0.306	0.776–2.245			
AFIP-Miettinen risk classification							
Very low-low risk versus moderate-high risk	10	2.374	0.001^*^	1.617–3.484	3.011	0.120	0.751–12.064
	5	2.368	0.001^*^	1.159–3.553	2.311	0.315	0.450–11.865

^*^ *P* < 0.05; HPF: high-power fields.
